# Balier’s French Narcissistic Theory of Ageing: Developments and Perspectives

**DOI:** 10.3389/fpsyg.2020.00113

**Published:** 2020-01-31

**Authors:** Franck Rexand-Galais, Johane Le Goff

**Affiliations:** ^1^BePsyLab and Spaces and Societies Research Team, Department of Psychology, University of Angers, Angers, France; ^2^Lucien Hussel Hospital, Vienne and Psychopathology and Clinical Psychology Research Centre, Lumière University Lyon 2, Lyon, France

**Keywords:** narcissism, ageing, depression, neurosis, ego ideal

## Abstract

Claude Balier’s narcissistic theory of ageing may be one of the pillars of French psycho-gerontology, but it is certainly not set in stone. On closer inspection, instead of being just minor variations on the same theme, the three stages of its development are structured around different references and make different assumptions. Some even contradict each other. Balier’s theory nevertheless finds unity in its non-involutionary view of ageing. Narcissism and narcissistic destiny are given a central role, thus calling into question the notion that the paradigmatic function of transference neurosis can account for neurosis in old age. For more than 40 years, Balier’s innovative notions have been the subject of many and sometimes divergent extensions in French psycho-gerontology.

## Introduction

The relationship between narcissism and ageing has primarily been explored in English-language publications over the past 25 years. Narcissism is increasingly being recognised as a protective factor in mental illness (Carter and Douglass, 2018), but its conceptualisation can vary, as can the methodologies used to explore it (Miller and Campbell, 2008). In France, when we talk about the French psychoanalytical theory of ageing, we immediately think of Claude Balier. His theory of narcissistic ageing was initially based on a clinical exploration of older individuals attending day centres, adopting a psychoanalytical approach. It has since been extensively developed in France, but remains unknown in the English-speaking world, which has consequently been unable to build on its explorations.

The Argentinian psychoanalyst Leopoldo Salvarezza also contributed to the narcissistic theory of ageing (Salvarezza, 1991, 2002), expounding similar ideas to those of Balier and sharing many references with him.

In 1979, Balier published an article in *L’Information Psychiatrique* entitled “Pour une théorie du vieillissement narcissique” [For a narcissistic theory of ageing]. This left such a mark on psychoanalytic research applied to old age that, according to François Villa, “all subsequent research has implicitly partaken in a secret dialogue with the pioneering work of Balier” (Villa, 2014, p. 23). It is well worth looking back at the genesis of this theory, which has made such an important contribution to the popularity of the narcissistic paradigm and called into question the relevance of the transference neurosis paradigm in ageing (Verdon, 2015). The texts published by Balier in 1976 (Balier, 1976b; Balier et al., 1976) make it easier to grasp the notions he subsequently put forward in 1979 and to see how they evolved into Balier’s final article on psycho-gerontology 3 years later: “Des changements de l’économie libidinale au cours du vieillissement” [Changes in the libidinal economy during ageing] (Balier, 1982). The narcissistic theory of ageing can be viewed as a plea for narcissism and the acknowledgement of its powerful role in ontogeny. This conceptualisation also supports the actual nature of neurosis in ageing. Without questioning this double paradigm, it is expressed through a flexible set of statements and references that reflects how Balier’s thinking was informed by his clinical practice with older individuals in France’s newly established day centres.

When a theory is not considered as a whole, it can give rise to variations between authors and their interpretations. This explains the diversity of perspectives that characterises the original approach to psycho-gerontology in French psychoanalysis.

## From Balier’s Early Works to the First Formulation of His Theory

Balier practised as a psychiatrist and as head of outpatient services between 1963 and 1965, at the Mental Health Association of the 13th district of Paris. While continuing to work as a psychiatrist, he then became the association’s head of psycho-gerontology, on the recommendation of two towering figures of French psychoanalysis, Philippe Paumelle and Serge Lebovici. This is when his gerontological activity took place. In 1977, he left the 13th district association to become head of the Regional Medical and Psychological Service at Varces Prison, near Grenoble (Balier and Lemaître, 2007). The article that instituted the narcissistic theory of ageing was therefore published 2 years after Balier had stopped working in gerontology. During his career as a gerontologist, Balier wrote fifteen or so texts on the specificities of the nascent field of gerontological psychiatry in France, focussing on the then little known population of older individuals receiving day care.

In 1976, the newly founded *Cahiers de la Fondation Nationale de Gérontologie* [Papers of the National Foundation of Gerontology], which later changed its name to *Gérontologie et Société*, gave Balier an opportunity to summarise his work in a special issue entitled *Individual Ageing and Social Ageing: For a Dynamic Approach to the Pathology of Senescence* (Balier et al., 1976). Balier penned two chapters of about a hundred pages: “Clinical study” (Balier, 1976b) and “Items for a narcissistic theory of ageing” (Balier, 1976a). These chapters were linked to another text, “Troubles névrotiques de la senescence” [Neurotic disorders of ageing] (Balier, 1976c), published the same year in the *Encyclopédie Médico-Chirurgicale*.

Balier made three initial contributions to the narcissistic theory of ageing. First, as part of his work he conducted within the Paris Psychoanalytical Society, of which he was a full member, he referred mainly to the important research on narcissism conducted by Béla Grunberger (1979). Since 1956, and therefore before Heinz Kohut (1977), Grunberger had highlighted and characterised the opposition within psychic life between the narcissistic dimension and the object-impulse dimension, between the “narcissistic sequence” and the “instinctual sequence” (Grunberger, 1979, p. 239). Balier also drew on the question of the self, developed by Kestemberg and Kestemberg (1966), which would also inform his explorations of violence some two decades later (Balier, 2005). Finally, he drew on works on depression, especially those by Nacht and Racamier (1959) and Francis Pasche (1963). North America was his second source of inspiration, for both psychoanalytic (e.g., Grotjahn, 1940; Kohut, 1977) and non-psychoanalytic (e.g., Cumming and Henry, 1961; Butler, 1963) references. As well as prompting reflection on unexplored epidemiological data in the French language and fostering the convergence of views (e.g., on the question of depression), these references allowed the role of social and identity factors in ageing to be considered. The third set of references was less homogenous. Indeed, Balier is part of a tradition that questions the involutionary approach to agieng and its pathological manifestations (Dedieu-Anglade, 1961), where it is viewed as a crisis (Erikson, 1959), and its cultural dimension is not necessarily considered (Dumazedier, 1974).

The 1976 texts should be considered as a single whole, particularly the two chapters in the *Cahiers*, which were constructed as mutual references. These texts set out a specific conception of narcissism and its role in ageing. They draw nosographic conclusions as to the narcissistic nature and currentness of pathology in ageing, and view depression in older individuals as having a fundamental function in the disintegration of the psyche.

The three 1976 texts agree on the definition of narcissism and its role in ageing: “We can define narcissism as self-investment, at the origin of the sense of identity, of self-confidence, of self-esteem” (Balier, 1976a, p. 131). According to Grunberger, this narcissistic self-investment is not conceived of in opposition to investment in external objects. It should be placed in a dynamic movement that mobilises the body, emotional bonds and the environment, especially in its social dimension. It is essential to take account of narcissism in ageing, as it brings “the feeling of inexorably moving toward death and naught” (Balier, 1976a, p. 133). Moreover, narcissism is defined as the necessary antidote to the abandonment of oneself to death and to a disinvestment associated with destruction, bearing in mind that when describing this fear of death, it is not necessary to refer to the drive or the death instinct (in French-language translations of Sigmund Freud, “Trieb” continued to be regularly translated as “instinct” rather than “drive” well into the 1980s). Ageing is therefore to be understood as a pendulum movement between self-investment and disinvestment. From this point of view, ageing is clearly a “problem of the economics of narcissism” (Balier, 1976a, p. 135).

The role attributed to narcissism in ageing necessarily has an impact on nosography. The most distinctive pathologies of old age, in particular depression and actual neurosis, can be linked to narcissistic disorders. They should not, therefore, be considered from an Oedipal point of view. Rather, they should be understood as belonging to what Grunberger described as “the narcissistic sequence, comprising narcissism, ego ideal, narcissistic injury, shame, loss of object anxiety, and depression” (Balier, 1976b, p. 118). The relative failure of the individual’s narcissism turns the actual neurosis into a psychosomatic reaction or psychosis - so much so that it can play a role in the development of dementia.

Depression therefore performs an organising function in these three texts, where there are no hermetic barriers between states. Depression should thus be understood as “a crossroads from which an individual may either continue into so-called *normal* ageing or enter a form of pathology” (Balier, 1976b, p. 119). For Balier, the prevalence of depression in ageing is underestimated. In reality, any change in ageing affects both identity and narcissism. Any modification leads to an impairment of self-esteem, and thus to a more or less intense depressive reaction. In this process, there are variations along three dimensions: body alteration, personal history, and the environment (which reflects a deficit image of ageing). This process can contribute to destructive narcissistic disinvestment.

The neuroses that appear with ageing are also the result of a depressive reaction, and stem from childhood psychic conflicts. Their effect can first be understood from what happens at the level of the bodily ego, which takes the place of the person’s ego ideal. The diminished body of old age is experienced by that person as a narcissistic injury - a sign of severance from either what they have been or the environment. The conflict therefore lies in the present. But there is more. In these 1976 texts, the ego ideal is just the third source of narcissism. The other two sources are primary narcissism (derived from primary maternal love) and the self. The self is not to be understood as the English-language self, in the sense of one’s own person, but in the tradition of the Kestembergs, as a differentiated entity possessing its own functional destiny, distinct from the ego and on which the narcissistic libido initially fastens itself. As ageing starts to affect both the ego and the ego ideal, the self takes over, promoting the pleasure (or even displeasure) of healthy functioning. There is an immediacy, a lack of distinction between subject and object, between inside and outside, and an investment in the proximal space that makes skin contact important. This regression can be experienced either negatively or positively, especially if the fear of loss is overcome. It is the central cause of actual neurosis, as it promotes the bodily reactivation and expression of possible primary experiences of displeasure. It is also the cause of many borderline signs and symptoms (lack of distinction between subject and object, etc.) in ageing, as Balier indicates, though without describing it as such in any of these three texts.

Finally, the 1976 texts deal with the current aspect of ageing pathologies. These are exclusively related to narcissism. Depression plays a central role in the destructuring of the psyche. The role of the self and its regression is also considered.

Dementia is only given a mention in the 1976 texts, but is more extensively discussed in the 1979 article.

## 1979: For a Narcissistic Theory of Ageing

“Pour une théorie narcissique du vieillissement” (Balier, 1979) was published in *L’Information Psychiatrique* 3 years after the 1976 texts. Balier had by now been working as a psychiatrist in a custodial setting for 2 years, but had not yet started to write about it, as his first important text on this type of clinical practice (“L’écoute des toxicomanes en prison” [Listening to drug addicts in jail]) was only published in Balier (1981).

Compared with the three 1976 publications, this article adopts a different approach to the narcissistic theory of ageing that can be divided into four components: (1) a vision of narcissism centred on the dimension of self-esteem; (2) an emphasis on the importance of the loss of choice of narcissistic object in the course of ageing-related depression and its repercussions on psychodynamic issues in dementia; (3) an approach to the neuroses of ageing centred on the question of the weakness of the ego; and (4) the possible link between the instinctual sequence and the narcissistic sequence.

Although the 1979 article does not primarily explore narcissism’s different theoretical dimensions, the final lines do make a distinction between narcissistic withdrawal, signalling pathological functioning, and narcissism that safeguards the self. There are very few references to Grunberger or, indeed, to any of the discussions of this issue within the Paris Psychoanalytical Society (Nacht, Racamier, etc.). Instead, it focuses on a rather narrow definition of narcissism that is exclusively oriented toward self-esteem. It is thus a legitimate extension of the 1976 texts. Narcissism as self-esteem emerges as a blueprint: “defined by Freud as the libidinal investment of the ego, and more clinically as self-esteem, narcissism is about the image we project of ourselves and that others reflect back” (Balier, 1979, p. 635). While narcissism remains the “guardian of life to the extent that it is a love of self” (Balier, 1979, p. 635), it is no longer embroiled in a struggle with the fear of death or any other destructive power. Instead, the article concentrates on the multifocal dimension of transformation and loss in the context of an ageing process that brings about biological but also environmental changes, not only from a social perspective (retirement, environment’s inability to meet community needs), but also within the family (e.g., widowhood, children leaving home) and in cultural representations (e.g., segregationism, negative representations of ageing).

The central function of depression as a crossroads is still present, but it is now associated with the question of the choice of a narcissistic object. Depression resulting from the loss of self-esteem, or “depression by narcissistic injury” (Balier, 1979, p. 637), remains the most common consequence of the generality of loss in old age. Balier now views it as the consequence of either the loss of an object chosen in a narcissistic modality, or a disturbance in the narcissistic sequence that cannot be attributed to the individual’s previous structure. This raises the question of the impact of transformations wrought by ageing on both the individual and his or her environment. The question of narcissism within dementia takes up far more room in the 1979 article than in the 1976 texts, and is linked to the question of the choice of narcissistic object, with a narcissistic dimension being assigned to object loss. Without questioning the degenerative dimension, Balier depicts the loss of the narcissistic object as a factor for dementia. He uses the famous manual published by Henri Ey, Paul Bernard and Charles Brisset in 1960 to support the psychodynamic dimension (disorganisation of the ego), and Freud to support the model of melancholy. Emphasising the importance of the depressive reaction (“the demented person is fundamentally depressed”; Balier, 1979, p. 641), he explores the specific role of the internalised maternal imago: “It is as if something of oneself, in relation to the internalised image of the mother, were attacked” (Balier, 1979, p. 642). This process leads to self-destruction in a situation of disjunction between the external reality and the internal object. As a result of linking the narcissistic and instinctual sequences, the internalised parental imago becomes a key issue in 1979.

Exploration of actual neuroses in ageing also leads to reformulations. In the 1976 texts, the reference to self is based on the Kestembergs. In the 1979 article, by contrast, Balier refers once again to Ey et al. (1960), and to Freud, in order to talk about manifestations that are thought to affect 33–35% of France’s ageing population, and which pave the way for more serious pathologies, mainly because of the depressive reaction they trigger. Actual neuroses can be attributed to the fact that the ego of ageing individuals has only limited capacities for adaptation and investment. This can arise from either the impossibility of releasing drive impulses in a situation of exaggerated ego-genicity (Freudian model of the fear of ageing) or a concentration of the libido on one of the parts of the body in the course of its transformation. Actual neuroses therefore raise the issue of narcissism because they reflect a failure to invest in objects. More so than in the 1976 explorations, the focus here is on the weakness of the ego (particularly the bodily ego) and on the complex and concomitant reworking of the object and narcissistic investment brought about by ageing. The 1979 narcissistic theory of ageing is more closely related to the instinctual sequence than that of 1976. It does not, however, see economic reorganisation in favour of narcissism as a regressive movement, but rather as a narcissistic destiny in a society characterised by restrictive cultural and environmental models.

Whereas the 1976 texts had devoted very little space to the instinctual sequence (Oedipus - superego - genital conflict - guilt - castration anxiety - neurotic symptoms), in the 1979 text, the connection between this sequence and the narcissistic sequence is now a given, as evidenced by the adjustments at different levels of the theory. This article asserts the importance of having a strong self and a strong Oedipal organisation to face up to the pangs of ageing, which reactivate castration anxiety. It is equally important to treat these pangs in a way that does not lead to disorganisation, even if, of course, “it is also understood that the continuation of identity through the vicissitudes of ageing requires good narcissistic investment” (Balier, 1979, p. 642) and, in the opposite case, “conventionally, the difficulties encountered open the way to regression on narcissistic bases” (Balier, 1979, p. 643). In its darker version, the narcissistic destiny is depicted as a consequence of the assaults of old age, as these assaults cause a failure of the instinctual sequence and thus a collapse of self-esteem. In its lighter version, this narcissistic destiny is conceived of as “covering all ages of life” (Balier, 1979, p. 644).

The 1979 article explores the conditions for a positive reorganisation of narcissism in old age. Balier considers the three sources of self-esteem from a Freudian perspective: primary narcissism (exploration of the self has completely disappeared), ego ideal, and object libido (absent from the 1976 texts). He states that, as the last two are affected by old age, common types of regressions can be expected. And yet North American authors (e.g., Butler) showed that a positive life balance can protect individuals from collapse. Balier retains the role of well internalised parental imagos in supporting identity and its continuation, as well as the individual’s relationship with the environment. In old age, the ego ideal aims to satisfy the desire to attune the environment with the repressed Oedipal representative, in particular by finding room for the idealised substitute object that occupied a significant place earlier in life (e.g., a friend who is now thought of every day) and which was able to satisfy the individual’s object and narcissistic libido: “Does the continuation of a satisfactory narcissism during ageing eventually cease to rely on acceptance of a lesser importance of the subject, in favour of being part of an environment that symbolises the satisfactory mother?” (Balier, 1979, p. 645). In the case of Oedipal maturation, a narcissistic balance emerges that is less dependent on secondary narcissism than on primary narcissism. The discovery of new relationships between the individual and his or her environment allows for a possible rebalancing of the ego ideal, geared toward finding a place in the generational chain - an aspect that Balier was to take further 3 years later.

## The Contributions of 1982: Changes in the Libidinal Economy During Ageing

“Des changements dans l’économie libidinale au cours du vieillissement,” the transcription of a lecture given in 1979 at the University of Lyon 2, was published in 1982. At the end of 1979, a group of Ph.D. students led by Jacques Gaucher had the idea of organising a conference entitled “Temporality and Ageing.” The proceedings, published the year that the publishing house Les Editions de La Chronique Sociale was founded, under the title *Le temps et la vie* [Time and Life] (Guillaumin and Reboul, 1982), was to constitute one of the cornerstones of French psycho-gerontological thought. Alongside Jean Bergeret, who gave a surprising lecture entitled “La deuxième crise d’adolescence. Sénescence et crise d’identité” [The second teenage rebellion. Ageing and identity crisis], and many of the thinkers belonging to the nascent movement of French psycho-gerontology, Balier presented the final chapter in the narcissistic theory of ageing. This lecture tackles two central issues: actual neuroses (revisited yet again), and positive narcissistic destiny. Regarding actual neuroses, it opens with an exploration of the anxiety that underlies the mid-life crisis. It describes the position set out by Freud (1895/1964) in “On the grounds for detaching a particular syndrome from neurasthenia under the description “anxiety neurosis,” whereby the fear of ageing is linked to an increase in somatic arousal that the psyche is unable to control. This postulate is extended to the question of actual neuroses in ageing, and ends with the conclusion that “whatever the force of the drive, psychic flow is often a problem” (Balier, 1982, p. 79). As in 1979, the focus seems to be on the ego, but this time it is the subject of detailed discussion. It is a decrease in the psychic libido that is responsible for them actual neuroses. The causes are twofold. The first is a flaw in the personality structure, namely a failure in the transition from primary narcissism to auto-eroticism (summoning up fantasies about the mother) and the development of a relationship between the latter and the forbidding figure of the stranger (prefiguring the father). During this Oedipal prefiguration, a vulnerability thus emerges, which may be related to a deficit in the mother’s ability to be absent or to be the lover (Braunschweig and Fain, 1975). In any event, this vulnerability has an impact on the ability to fantasise, and affects the deployment of the Oedipus. The second cause is the reworking of the ego ideal. In order for it to be reactivated during ageing, it needs to revive itself in primary narcissism and thus produce a structuring regression. However, this is impossible for some, owing to a failure in their primary narcissism: “the symptom here blocks the way of the distressing representation linked to the desire for regression, which hides the depressive emptiness” (Balier, 1982, p. 85). The disappearance of psychic life in actual neuroses therefore corresponds to a psychic death that has its counterpart in dementia, through the dissolution of the ego. This results mainly from a gap between the ego and the ego ideal. In the case of dementia, it is the gradual transformation of unbound aggression into self-destruction, activating the portion of self-destruction (death instinct) inside the individual, that ultimately kills the latter, in accordance with the Freudian thinking contained in “An outline of psycho-analysis” (Freud, 1939/1962) and taken up by Balier. In the absence of deterioration, the identification with death becomes too great under the effects of old age, and may thus make revival in primary narcissism (and thus reunion with the maternal object) through the ego ideal impossible. The result is a loss of symbolism and fantasy life in favour of the external reality, this being the “manifestation of the death instinct” (Balier, 1982, p. 86).

The lecture also deals with persons whose successive losses and bereavements have failed to dent the narcissistic investment of their ego. These are individuals whose narcissism has a positive destiny. The data that Balier analysed were collected from retired teachers aged over 85 years. He observed a strong Oedipal organisation, a narcissistic investment of the operating ego, and an unaffected ego ideal. With this shift in interest toward a general population in good mental health, the conceptual approach to narcissism changed yet again: “we use it here in the sense of a personal feeling of value, the basis of self-confidence, which the self needs to show interest in others. Self-investment is also the source of the sense of identity: to exist among others” (Balier, 1982, p. 80). The deconstructing function of depression, so pervasive in the 1976 and 1979 texts, is, in effect, erased here. In this population, Balier observed an ego ideal that is not necessarily anchored in the past and which allows the ego to maintain its coherence. Thus, a positive life assessment is not the only factor involved in the sense of coherence. In these individuals, an active ego ideal is preserved, and there is a “prerogative in relation to the superego” (Balier, 1982, p. 82), testifying to the fact that “the narcissistic sequence prevails over the instinctual sequence” (Balier, 1982, p. 83). This perspective, however, is immediately challenged: “to tell the truth, it is rather a new balance between the superego and ideal ego” (Balier, 1982, p. 83). This is because a solid superego allows the individual to remain strong in the face of losses, and not to experience these losses in terms of castration. Ideals and identifications stemming from the narcissistic sequence also contribute to this balance. In its positive destinies, the narcissistic theory of ageing is therefore also a genital-narcissistic theory.

The 1982 text therefore deals with two main issues: the problem of drive flow and its causes (failure of primary narcissism and reworking of the ego ideal), and the exploration of positive narcissism in a population in good mental health, where the Oedipal and narcissistic organisations work in a balanced way.

## Discussion

### Questioning the Unity of the Narcissistic Theory of Ageing

Narcissism is clearly defined by Balier (1976, 1979, 1982) as self-esteem. It is approached according to its different destinies in ageing: depression, dementia, and fear of ageing. These destinies are considered in the light of several modalities: choice of narcissistic object, internalised parental imago, instinctual and narcissistic sequences, and the rearrangement of the different psychic entities.

Describing the gestation of Balier’s narcissistic theory of ageing provides a means of exploring both the unity and diversity of the paths down which his thinking led him. For example, in the case of actual neuroses, the idea of a failure of primary narcissism is a constant, but the way in which it is situated is formulated differently at each period. Balier seems to seek the explanation that is most in line with his current clinical practice, whether it be with the populations encountered in day centres or those with a high sociocultural level. Sometimes, the outcome is surprising. For instance, fear of death seemed extremely important in 1976, but was replaced in 1979 by the effects of multiple losses experienced in terms of castration. Then, in 1982, a reference was made to the death instinct, which had previously been rejected as a conceptual solution. These variations are sometimes discordant and complex, making it difficult to present the theory as a consistent whole. As a result, authors sometimes reduce it to its lowest common denominators. As Charazac complains, “The narcissistic theory of ageing is commonly presented as an assembly of negative images or *snap-shots*, where the terms of attack, injury and loss coexist with the notions of shoring up, reassurance and ageing well” (Charazac, 2014, p. 309). These potentially impoverishing differences are also signs of wealth, which probably explains why there is also a tendency to retain or discuss a single version of this theory. It is not always easy to choose one version over another, and in any case one only has to read the discussions about this *luminous* theory (Dibie-Racoupeau and Granet, 2010) to realise just how consistent it is when it comes to, say, tackling questions as diverse as neurotic suffering in older people (Verdon, 2004a), creativity (Talpin, 2011) or the mid-life crisis (Péruchon, 2008). Recent explorations based on the issue of retirement (Le Goff and Rexand-Galais, 2018) also show how different parts of the theory can be linked together and discussed.

The apparent discrepancies can also be viewed as the marks of a constant quest for adjustment. Thus, whereas the instinctual and narcissistic sequences are set against each other in the 1976 texts, they are connected in the 1982 lecture, where the destinies of narcissism are expressed in their diversity. This lecture explores the conditions favouring successful destinies of genital narcissism, whereas the 1976 texts consider the conditions favouring the collapse of this instinctual sequence and the expression of destructive narcissism. In this regard, the article published in 1979 in *L’Information Psychiatrique* bridges the gap between the two, and is certainly the one that best describes the toing and froing between the instinctual and narcissistic sequences in ageing. While the theory was formulated and reformulated, it is its unity, and not its points of divergence, that should be underscored. The various types of care that are now available for older individuals are certainly moving in this direction.

### Four Extensions of the Narcissistic Theory of Ageing in France Since C. Balier

Since Balier’s day, the narcissistic theory of ageing has undergone four types of extension: (1) an exploration on the basis of theoretical insights that were not used by Balier; (2) an exploration applying psychotherapeutic approaches, mainly centred on narcissism in ageing and narcissistic restoration; (3) an exploration of clinical situations that Balier generally did not consider in his theorising; and (4) an exploration aimed at achieving a new balance, by reintroducing the object axis. Considerable time passed before the narcissistic theory of ageing started to be reinterpreted on the basis of a psychoanalytic epistemology foreign to that of the Paris Psychoanalytical Society. In fact, 20 years went by before Lacan’s reflection on narcissism encountered Balier’s theory. Balier’s references were by no means Lacanian. In 1992, Jack Messy (1992), who also worked in the day centre of the 13th district of Paris, took many of Balier’s proposals and explored them again in a Lacanian light. In *La personne âgée n’existe pas* [The elderly person does not exist], Messy put the Lacanian mirror stage back at the heart of ontogenesis. He reminded us in particular of the child’s role as a mediator of the ego ideal in an aggressive tension between the ego and the ideal ego, as much as his unifying function. As individuals move into their fifties and sixties, they encounter another test of the mirror - that of the *broken mirror*. This fresh encounter has none of the jubilation of the one in childhood. Instead, it has a distressing dimension, constituting the “anticipation, in the mirror or in the image of another older person, of one’s own image in death” (p. 81). The ego ideal collapses as a *self-hideousness* manifests itself, and with this fall, the aggressive tension plays a regulating role. The self, alienated by this hideous image, is then confronted with strangeness and fragmentation, while the projection of an addiction onto the other is organised as a new alienation of the ego. This reignites the Kleinian depressive position, thus activating neurotic or perverse moments and, of course, a process of reorganisation. For Messy, when the narcissistic foundations are so fragile, there is a high risk of a “new loss of an invested object” occurring, “whose mourning is not possible, but which will transform the event into a trauma, without doubt in relation to an original trauma, not symbolised” (p. 82). It is this experience of an “undue loss” (e.g., death, theft, loss of ability) that pushes individuals into depression, as this test results in the object libido being pulled back into the ego or the body. As in Balier, therefore, old age is not inevitable. This means that the ego ideal has to be reconfigured, as its social dimension rests on data that generally depict old age as involutionary, meaning that ageing individuals are not valued. There is therefore a high risk of failure during the psychic remodelling. Like Balier, Messy put narcissism at the heart of pathology (e.g., in dementia, the autolysis of the ego, following the breaking of the mirror and the erasure of the symbolism, plays an important role). Through Messy, Balier was now linked to Lacanian theorisation. The narcissistic theory of ageing was set to spread in the French field without its founder necessarily being cited, given the increasing references to Messy.

Explorations of the psychotherapy of narcissism in ageing and the conditions of narcissistic restoration began in Balier’s wake. It is interesting to note that Balier himself, through his work on the psychotherapeutic treatment of violent behaviour, contributed to the question of narcissistic restoration, by combining a psychoanalytic referent and a psycho-corporeal approach centred mainly on relaxation (Balier, 2003). Generally acknowledging the need for a psycho-corporeal approach, be it relaxation or, say, sophrology, these explorations (Langendorff, 2004; Rexand-Galais, 2013; Le Goff and Rexand-Galais, 2018) stress the need for the framework to be adapted. It is often assumed that this framework needs to be warmer, and the sessions both shorter and more frequent, to foster the revival of psychic life and the restoration of the narcissistic foundations (mainly of the ego ideal) favouring the object axis and, ultimately, stimulating the individual’s relational appetite. As narcissistic withdrawal into the body is exacerbated by ageing, and as free association is often delicate (with individuals for whom ageing has been a time of separation from others as well as from the self), these explorations also make it possible to observe the value of psycho-corporal mediation in the psychotherapy of older individuals. This mediation fosters a new relationship with the body, mobilises the imaginary function, and helps individuals revisit their experiences, by producing a new subjectivity in the face of past events (Fromage, 2001). This psychotherapeutic approach to the care of older persons has become increasingly popular, especially in institutions.

The narcissistic theory of ageing highlights clinical aspects that deserve further exploration. It is particularly important to think about borderline personality in ageing. The narcissistic theory can help to shed light on a “shared borderline disorganisation in ageing” and analyse its different destinies (Rexand-Galais, 2019). Although the narcissistic theory of ageing is not objectively referenced, it can support the psychotherapeutic work undertaken with older persons. It is particularly relevant when it comes to the mourning of the ego, or the working of ageing (Bianchi, 1980), both very common in the ageing process. The narcissistic theory of ageing is a valuable tool for softening “the mirror of a failing narcissism” (Bloch et al., 2008, p. 64), so that the body becomes the source of the pleasure of functioning. This helps to restore, revive and revitalise narcissism. In addition, as with the psychotherapeutic assistance given to ageing individuals, Balier’s narcissistic theory of ageing is key to rehabilitating an overdemanding ego ideal and the internal elaboration of life’s trials (Racin, 2015). In his work on parental imagos, Pierre Charazac (2018) underlines the importance of these roles in the “work of dying” (De M’Uzan, 2013, p. 33), for both the patient and the therapist. The transformational capacity of parental imagos can effectively support this work, allowing the object libido and narcissistic libido to be balanced. Marion Péruchon (2013) recently reviewed the notion of resilience, as discussed by Boris Cyrulnik and Duval (2006), and in particular the importance of positive narcissism in the adage of narcissism, mentalisation, and object/environment. She reminds us that “narcissism, among other things, supports resilience, especially in the course of ageing, which exposes the self to multiple traumas” (2006, p. 76). The narcissistic theory of ageing thus addresses the role of narcissism in the unification of the ego, which relies on primary narcissistic foundations. It also evokes Cyrulnik (2008)’s notion of “custodian of resilience” (p. 152), reminiscent of the notion of internalised object and thought to fulfil a similar function to Wilfred Bion’s alpha function (Bion, 1967).

The narcissistic theory of ageing can also be used to explore the reconciliation between object and narcissistic issues. Benoît Verdon states that Balier’s paradigm of actual neurosis “has proved to be a privileged paradigm for thinking about the specificity of the neurotic psychopathology of ageing, to the detriment of the paradigm of the transference neurosis, and the place of the sexual dimension and a complex internal temporality” (2015, p. 130). Nevertheless, given the multifaceted nature of clinical practice among older individuals, Verdon emphasises the need to draw links between the narcissistic axis and the object axis (Verdon, 2004b) and to remember that some pathologies of ageing belong strictly to the Oedipal register. Thus, the issue of the object and narcissistic dimensions is regularly discussed among French gerontologists, as are the paradigms of current neurosis and transference neurosis (Verdon, 2015; Charazac, 2018).

Whereas Paul-Laurent Assoun (1983) reasserted the importance of the choice of narcissistic object for ensuring psychic continuity, Verdon (2015) foregrounds the structuring aspect of new object modalities and the importance of the forbidden. More recently, Tapia and Péruchon (2018) alluded to the linking of the narcissistic and instinctual sequences when they discussed the work of mourning that is so present in older individuals. They see this work as the need for individuals to separate themselves from their narcissism whilst simultaneously introducing good objects. Narcissism then recharges itself libidinally. Céline Racin (2015) also stresses the need to balance the early relations assigned to the Oedipal register and the crises of ageing. The fall and the current narcissistic upheaval bring the Oedipal conflict back to the forefront. Here, the link between early relationships and the crises currently experienced by older people seems essential.

The issue of Oedipal work, especially its connection to the compulsion of repetition and the death instinct, emerges afresh when we discuss the link between past and present history. Michel De M’Uzan (2017) worked on the concept of death instinct, based on the distinction between the same and the identical. According to him, personality goes in two directions, depending on “The existence or not of a solid elaboration of the category of the past” (De M’Uzan, 2017, p. 29). Thus, in persons who have worked out the Oedipus, the story is reprocessed in terms of castration problems (i.e., it is replayed in the same way). This is the classic transference neurosis. By contrast, individuals with a flawed Oedipal elaboration present a clinical picture of repetition compulsion (i.e., repetition of the identical).

D’Muzan’s theoretical elaboration allows us to think about repetition compulsion in terms of transfer modalities and topical interactions. The death instinct has also been considered in the work of psychosomatics. This notion makes it possible to grasp the construction of pathological structures of the personality according to the death instinct (De Masi, 2015). The latter is now moving toward the notion of destructiveness, which is extensively used in France (Péruchon, 2014).

## Conclusion

Claude Balier’s narcissistic theory of ageing mainly focuses on the dimension of narcissism. It espouses a non-regressive view of ageing, and calls into question the transference neurosis paradigm. It also affords considerable importance to the economic dimension. This theory has had a considerable impact on how narcissism is viewed in older individuals, and its various reworkings and reformulations indicate the diversity of its scope, in a world where changing living conditions and a longer life expectancy entail an increasingly diversified clinical practice with seniors. Even if the different parts of the theory reflect a multiplication of clinical situations that emphasises its relevance, the issue of narcissism in particular seems pivotal to current clinical issues.

Psychotherapeutic approaches are thus determined by the narcissistic issues that are currently faced by individuals. Clinicians must then decide whether to approach the Oedipal issue.

## Author Contributions

Both authors listed have made a substantial, direct and intellectual contribution to the work, and approved it for publication.

## Conflict of Interest

The authors declare that the research was conducted in the absence of any commercial or financial relationships that could be construed as a potential conflict of interest.
